# Supplementary material for: Prevalence and Spectrum of Second Primary Malignancies among People Living with HIV in the French Dat’AIDS Cohort
Source: Cancers (Basel). 2022 Jan 13;14(2):401. doi: 10.3390/cancers14020401 (PMC8773756; doi:10.3390/cancers14020401)
Supplement: Supplementary file 1 [file cancers-14-00401-s001.zip › Supplemental Table S1.pdf]

Supplemental Table S1: Pattern of first and second primary cancer types in the French Dat'AIDS cohort

ICD-10 Codes	First primary cancer types	ICD-10 Codes	Second primary cancer types	Females N= 75	Males N=369	Total N= 444
	ADCs			***32 (42·7)***	***237 (64·2)***	***269 (60·6)***
C46	Kaposi sarcoma			7 (9·3)	149 (40·4)	156 (35·1)
			Non- HPV oral cavity/pharynx:	0 (0·0)	5 (1·3)	5 (1·1)
		C00	- Lip	0	2	2
		C03	- Gum	0	1	1
		C06	- Mouth, other location not specified	0	1	1
		C07	- Parotid	0	1	1
			HPV- related oral cavity/pharynx	1 (1·3)	2 (0·5)	3 (0·7)
		C02	- Tongue, other location not specified	0	1	1
		C09	- Tonsil	0	1	1
		C14	- Lip, oral cavity and pharynx locations poor specified	1	0	1
		C15	Esophagus	0	1	1
		C16	Stomach	0	1	1
		C17	Small intestine	0	2	2
		C18	Colon	0	4	4
		C20	Rectum	0	5	5
		C21	Anus	0	13	13
		C22	Liver and intrahepatic bile duct	0	4	4
		C24	Extrahepatic bile duct	0	1	1
		C30	Nasal cavity and middle ear	0	2	2
		C34	Lung and bronchial	2	4	6
		C38	Pleura/mediastinum/heart	0	1	1
		C40	Bone and joints	0	1	1
		C41	Bone/joint, others location not specified	0	1	1
		C43	Melanoma	0	3	3
		C44	Skin carcinoma	1	22	23
		C50	Breast	0	1	1
		C60	Penis	NA	1	1
		C61	Prostate	NA	8	8
		C64	Kidney without pelvis	0	1	1
		C66	Upper urinary tract	0	1	1
		C67	Urinary bladder	0	1	1
		C76	Other locations and poor specified	0	2	2
		C81	Hodgkin lymphoma	0	11	11
			Non-Hodkin lymphoma	3 (4·0)	47 (12·7)	50 (11·3)
		C82	- Follicular lymphoma	0	1	1
		C83	- Diffuse large B- cell lymphoma	0	13	13
		C84	- NK/T cell Lymphoma	0	3	3
		C85	- Others lymphoma not specified	3	30	33
		C90	Myeloma and plasmacytoma	0	1	1
		C92	Myeloid leukemia	0	1	1
		C94	Others leukemia with cells specified	0	2	2
	Non- Hodgkin lymphoma			17 (22·6)	88 (23·8)	105(23·6)
C82	Follicular lymphoma			1 (1·3)	0 (0·00)	1 (0·2)
		C34	Lung and bronchial	1	0	1
C83	Diffuse large B- cell lymphoma			3 (4·0)	27 (7·3)	30 (6·7)
		C02	Tongue, other location not specified	0	1	1
		C10	Oropharynx	0	1	1
		C20	Rectum	0	1	1
		C22	Liver and intrahepatic bile duct	0	2	2
		C25	Pancreas	1	0	1
		C30	Nasal cavity and middle ear	1	0	1
		C34	Lung and bronchial	0	4	4
		C44	Skin carcinoma	0	3	3
		C46	Kaposi sarcoma	0	6	6
		C61	Prostate	NA	1	1
		C81	Hodgkin lymphoma	0	3	3
		C85	Others lymphoma not specified	0	5	5
		C96	Histiocytosis/ histiocytic lymphoma	1	0	1
C84	NK/T cell Lymphoma			0 (0·0)	1 (0·3)	1 (0·2)
		C26	Digestive organs, others locations poorly specified	0	1	1

C85	Others lymphoma not specified			13 (17·3)	60 (16·3)	73 (16·4)
		C01	Base of the tongue	0	1	1
		C09	Tonsil	0	1	1
		C15	Esophagus	0	1	1
		C16	Stomach	0	1	1
		C21	Anus	0	4	4
		C22	Liver and intrahepatic bile duct	2	2	4
		C25	Pancreas	1	0	1
		C34	Lung and bronchial	1	2	3
		C44	Skin carcinoma	1	3	4
		C46	Kaposi sarcoma	2	17	19
		C50	Breast	3	0	3
		C61	Prostate	NA	1	1
		C62	Testis	NA	1	1
		C64	Kidney without pelvis	0	3	3
		C66	Upper urinary tract	0	1	1
		C67	Urinary bladder	0	1	1
		C72	Spinal cord/cranial nerves	0	1	1
		C81	Hodgkin lymphoma	1	6	7
		C83	Diffuse large B- cell lymphoma	2	10	12
		C91	Lymphoid leukemia	0	1	1
		C92	Myeloid leukemia	0	3	3
C53	Cervix			8 (10·7)	NA	8 (1·8)
		C21	Anus	1		1
		C22	Liver and intrahepatic bile duct	1		1
		C44	Skin carcinoma	3		3
		C46	Kaposi sarcoma	1		1
		C49	Sarcoma/connective tissu	1		1
		C50	Breast	1		1
	VR-NADCs			***6 (8·0)***	***45 (12·2)***	***51 (11·5)***
C21	Anus			2 (2·7)	12 (3·2)	14 (3·1)
		C02	Tongue, other location not specified	0	1	1
		C03	Gum	0	1	1
		C09	Tonsil	0	1	1
		C18	Colon	0	1	1
		C19	Rectosigmoid junction	1	0	1
		C20	Rectum	0	1	1
		C43	Melanoma	0	1	1
		C44	Skin carcinoma	0	1	1
		C46	Kaposi sarcoma	0	1	1
		C49	Sarcoma/connective tissu	1	0	1
		C50	Breast	0	1	1
		C61	Prostate	NA	1	1
		C81	Hodgkin lymphoma	0	1	1
		C84	NK/T cell Lymphoma	0	1	1
C22	Liver and intrahepatic bile duct			0 (0·0)	9 (2·4)	9 (2·03)
		C06	Mouth, other location not specified	0	1	1
		C16	Stomach	0	1	1
		C22	Liver and intrahepatic bile duct	0	1	1
		C25	Pancreas	0	1	1
		C32	Larynx	0	1	1
		C34	Lung and bronchial	0	1	1
		C83	Diffuse large B- cell lymphoma	0	2	1
		C92	Myeloid leukemia	0	1	1
C81	Hodgkin lymphoma			2 (2·7)	14 (3·8)	16 (3·6)
		C19	Rectosigmoid junction	0	1	1
		C21	Anus	0	1	1
		C22	Liver and intrahepatic bile duct	1	0	1
		C34	Lung and bronchial	0	1	1
		C44	Skin carcinoma	0	1	1
		C46	Kaposi sarcoma	0	1	1
		C51	Vulva	1	NA	1
		C73	Thyroid	0	1	1
		C83	Diffuse large B- cell lymphoma	0	5	5
		C85	Others lymphoma not specified	0	2	2
		C92	Myeloid leukemia	0	1	1
	HPV- related oral cavity/pharynx (C02;C09;C014)			*0 (0·0)*	*9 (2·4)*	*9 (2·0)*
C02	Tongue, other location not specified				4 (1·1)	4 (0·9)
		C01	Base of the tongue	0	3	3

		C34	Lung and bronchial	0	1	1
C09	Tonsil				4 (1·1)	4 (0·9)
		C02	Tongue, other location not specified	0	1	1
		C22	Liver and intrahepatic bile duct	0	1	1
		C34	Lung and bronchial	0	1	1
		C67	Urinary bladder	0	1	1
C14	Lip, oral cavity and pharynx locations poor specified				1 (0·3)	1 (0·2)
		C85	Others lymphoma not specified	0	1	1
C60	Penis	C18	Colon	NA	1 (0·3)	1 (0·2)
C51	Vulva			2 (2·7)	NA	2 (0·4)
		C21	Anus	1	0	1
		C50	Breast	1	0	1
	VU-NADCs			***37 (49·3)***	***87 (23·6)***	***124 (27·9)***
C03	Gum			0 (0·0)	2 (0·5)	2 (0·4)
		C34	Lung and bronchial	0	1	1
		C41	Bone/joint, others location not specified	0	1	1
C04	Floor of the mouth			0 (0·0)	1 (0·3)	1 (0·2)
		C46	Kaposi sarcoma	0	1	1
C05	Palate			0 (0·0)	2 (0·5)	2 (0·4)
		C00	Lip	0	1	1
		C44	Skin carcinoma	0	1	1
C07	Parotid			0 (0·0)	1 (0·3)	1 (0·2)
		C85	Others lymphoma not specified	0	1	1
C11	Rhinopharynx			1 (1·3)	0 (0·0)	1 (0·2)
		C14	Lip, oral cavity and pharynx locations poor specified	1	0	1
C12	Pyriform sinus			0 (0·0)	1 (0·3)	1 (0·2)
		C10	Oropharynx	0	1	1
C16	Stomach			0 (0·0)	1 (0·3)	1 (0·2)
		C15	Esophagus	0	1	1
C18	Colon			1 (1·3)	1 (0·3)	2 (0·4)
		C16	Stomach	0	1	1
		C19	Rectosigmoid junction	1	0	1
C19	Rectosigmoid junction			1 (1·3)	0 (0·0)	1 (0·2)
		C43	Melanoma	1	0	1
C20	Rectum			2 (2·7)	2 (0·5)	4 (0·9)
		C19	Rectosigmoid junction	1	0	1
		C21	Anus	1	0	1
		C61	Prostate	NA	1	1
		C64	Kidney without pelvis	0	1	1
C25	Pancreas			0 (0·0)	1 (0·3)	1 (0·2)
		C83	Diffuse large B- cell lymphoma	0	1	1
C32	Larynx			1 (1·3)	3 (0·8)	4 (0·9)
		C22	Liver and intrahepatic bile duct	1	0	1
		C34	Lung and bronchial	0	1	1
		C44	Skin carcinoma	0	1	1
		C61	Prostate	NA	1	1
C34	Lung and bronchial			1 (1·3)	11 (3·0)	12 (2·7)
		C18	Colon	0	2	2
		C21	Anus	0	1	1
		C22	Liver and intrahepatic bile duct	0	1	1
		C30	Nasal cavity and middle ear	0	1	1
		C32	Larynx	0	1	1
		C34	Lung and bronchial	0	2	2
		C43	Melanoma	0	1	1
		C50	Breast	1	0	1
		C83	Diffuse large B- cell lymphoma	0	1	1
		C85	Others lymphoma not specified	0	1	1
C38	Pleura/mediastinum/heart			0 (0·0)	1 (0·3)	1 (0·2)
		C34	Lung and bronchial	0	1	1
C40	Bone and joints			0 (0·0)	1 (0·3)	1 (0·2)
		C85	Others lymphoma not specified	0	1	1
C41	Bone/joint, others location not specified			0 (0·0)	1 (0·3)	1 (0·2)
		C83	Diffuse large B- cell lymphoma	0	1	1
C43	Melanoma			0 (0·0)	4 (1·1)	4 (0·9)
		C44	Skin carcinoma	0	2	2
		C84	NK/T cell Lymphoma	0	2	2
C44	Skin carcinoma			5 (6·7)	18 (4·9)	23 (5·0)
		C00	Lip	0	1	1

		C09	Tonsil	0	1	1
		C18	Colon	0	1	1
		C20	Rectum	0	1	1
		C21	Anus	1	0	1
		C22	Liver and intrahepatic bile duct	0	1	1
		C32	Larynx	0	1	1
		C34	Lung and bronchial	0	3	3
		C38	Pleura/mediastinum/heart	0	1	1
		C43	Melanoma	2	2	4
		C46	Kaposi sarcoma	1	1	2
		C53	Cervix	1	NA	1
		C61	Prostate	NA	3	3
		C76	Other locations and poor specified	0	1	1
		C80	Cancer location not specified	0	1	1
C49	Sarcoma/ Connective tissu			2 (2·7)	0 (0·0)	2 (0·4)
		C43	Melanoma	1	0	1
		C55	Uterus, not specified	1	NA	1
C50	Breast			12 (16·0)	0 (0·0)	12 (2·7)
		C34	Lung and bronchial	2	0	2
		C46	Kaposi sarcoma	1	0	1
		C50	Breast	4	0	4
		C51	Vulva	1	0	1
		C53	Cervix	1	0	1
		C54	Uterus corpus	1	0	1
		C70	Meninge	1	0	1
		C80	Cancer location not specified	1	0	1
C55	Uterus, not specified			1 (1·3)	NA	1 (0·2)
		C50	Breast	1		1
C56	Ovarian			3 (4·0)	NA	3 (0·7)
		C22	Liver and intrahepatic bile duct	1		1
		C44	Skin carcinoma	1		1
		C69	Eye and ocular annexes	1		1
C57	Genital organs, others not specified, females			1 (1·3)	NA	1 (0·2)
		C76	Other locations and poor specified	1		1
C61	Prostate			NA	10 (2·7)	10 (2·2)
		C16	Stomach		2	2
		C20	Rectum		1	1
		C21	Anus		1	1
		C34	Lung and bronchial		1	1
		C43	Melanoma		1	1
		C44	Skin carcinoma		1	1
		C46	Kaposi sarcoma		1	1
		C67	Urinary bladder		2	2
C63	Genital organs, others not specified, males			NA	1 (0·3)	1 (0·2)
		C34	Lung and bronchial		1	1
C64	Kidney without pelvis			0 (0·0)	10 (2·7)	10 (2·2)
		C21	Anus	0	1	1
		C33	Trachea	0	1	1
		C34	Lung and bronchial	0	1	1
		C44	Skin carcinoma	0	2	2
		C61	Prostate	NA	1	1
		C67	Urinary bladder	0	1	1
		C74	Adrenal	0	1	1
		C83	Diffuse large B- cell lymphoma	0	1	1
		C85	Others lymphoma not specified	0	1	1
C67	Urinary bladder			0 (0·0)	4 (1·1)	4 (0·9)
		C16	Stomach	0	1	1
		C18	Colon	0	1	1
		C46	Kaposi sarcoma	0	1	1
		C61	Prostate	NA	1	1
C68	Urinary organ others not specified			0 (0·0)	1 (0·3)	1 (0·2)
		C18	Colon	0	1	1
C69	Eye and ocular annexes			0 (0·0)	1 (0·3)	1 (0·2)
		C41	Bone/joint, others location not specified	0	1	1
C71	Brain[‡]			0 (0·0)	1 (0·3)	1 (0·2)
		C34	Lung and bronchial	0	1	1
C73	Thyroid			3 (4·0)	2 (0·5)	5 (1·1)
		C38	Pleura/mediastinum/heart	0	1	1
		C50	Breast	1	0	1

		C56	Ovarian	1	NA	1
		C81	Hodgkin lymphoma	1	0	1
		C83	Diffuse large B- cell lymphoma	0	1	1
C80	Cancer location not specified			2 (2·7)	2 (0·5)	4 (0·9)
		C04	Floor of the mouth	0	1	1
		C44	Skin carcinoma	1	0	1
		C46	Kaposi sarcoma	1	1	2
C90	Myeloma and plasmacytoma			0 (0·0)	4 (1·1)	4 (0·9)
		C22	Liver and intrahepatic bile duct	0	1	1
		C25	Pancreas	0	1	1
		C46	Kaposi sarcoma	0	1	1
		C79	Secondary malignant tumor with location not specified	0	1	1
C94	Others leukemia with cells specified			1 (1·3)	0 (0·0)	1 (0·2)
		C32	Larynx	1	0	1

Abbreviations: ADCs: AIDS-defining cancers; AIDS: acquired immunodeficiency syndrome; HPV: human papillomavirus; ICD-10: international statistical classification of diseases and related health problems, tenth revision; VRNADCs: virus- related non-AIDS- defining cancers; VUNADCs: virus-unrelated non-AIDS- defining cancers. NK/T: lymphocytes natural killer T

‡ This category does not include central nervous system lymphoma (CNS NHL)
