# Supplementary material for: Prevalence and Spectrum of Second Primary Malignancies among People Living with HIV in the French Dat’AIDS Cohort
Source: Cancers (Basel). 2022 Jan 13;14(2):401. doi: 10.3390/cancers14020401 (PMC8773756; doi:10.3390/cancers14020401)
Supplement: Supplementary file 1 [file cancers-14-00401-s001.zip › Supplemental Table S2.pdf]

Supplemental Table S2. Pattern of SPCs* according to the primary cancer among WLWH** in the French Dat'AIDS Cohort

First primary cancer types	N (%)	Second primary cancer types	N
NHL*** (C82, C83, C85)	17 (22.6)	C50- Breast	3
		C22- Liver and intrahepatic bile duct	2
		C25- Pancreas	2
		C34- Lung and bronchial	2
		C46- Kaposi sarcoma	2
		C83- Diffuse large B- cell lymphoma	2
		C30- Nasal cavity and middle ear	1
		C44- Skin carcinoma	1
		C81- Hodgkin lymphoma	1
		C96- Histiocytosis/ histiocytic lymphoma	1
Breast cancer (C50)	12 (16.0)	C50- Breast	4
		C34- Lung and bronchial	2
		C46- Kaposi sarcoma	1
		C51- Vulva	1
		C53- Cervix	1
		C54- Uterus corpus	1
		C70- Meninge	1
		C80- Cancer location not specified	1
Cervical cancer (C53)	8 (10.7)	C44- Skin carcinoma	3
		C21- Anus	1
		C22- Liver and intrahepatic bile duct	1
		C46- Kaposi sarcoma	1
		C49- Sarcoma/connective tissu	1
		C50- Breast	1
Kaposi sarcoma (C46)	7 (9.3)	C85- Others lymphoma not specified	3
		C34- Lung and bronchial	2
		C14- Lip, oral cavity and pharynx locations poor specified	1
		C44- Skin carcinoma	1
Females cancers (including C50, C53)	27 (36.0)		
- Vulva cancer (C51)	2	C21- Anus	1
		C50- Breast	1
- Uterus cancer (C55)	1	C50- Breast	1
- Ovarian (C56)	3	C22- Liver and intrahepatic bile duct	1
		C44- Skin carcinoma	1
		C69- Eye and ocular annexes	1
-Genital organs, others not specified, females (C57)	1	C76- Other locations and poor specified	1

Abbreviations: * SPC:Second primary cancers, ** WLWH: Women living with HIV; *** NHL: Non Hodgkin lymphoma
