# Supplementary material for: Prevalence and Spectrum of Second Primary Malignancies among People Living with HIV in the French Dat’AIDS Cohort
Source: Cancers (Basel). 2022 Jan 13;14(2):401. doi: 10.3390/cancers14020401 (PMC8773756; doi:10.3390/cancers14020401)
Supplement: Supplementary file 1 [file cancers-14-00401-s001.zip › Supplemental Table S3.pdf]

Supplemental Table S3. Pattern of SPCs¥ according to the primary cancer among MLWHβ in the French Dat'AIDS Cohort

First primary cancer types	**N (%)**	Second primary cancer types	N
Kaposi sarcoma	149 (40.4)	C82-C85- NHL*	47 (31.5)
		C44- Skin carcinoma	22 (14.8)
		C21- Anus	13 (8.7)
		C81- Hodgkin lymphoma	11 (7.4)
		C61- Prostate	8
		C18-C20- Colon/rectum	9
		C22- Liver and intrahepatic bile duct	4
		C34- Lung and bronchial	4
		C43- Melanoma	3
		C00- Lip	2
		C17- Small intestine	2
		C30- Nasal cavity and middle ear	2
		C76- Other locations and poor specified	2
		C94- Others leukemia with cells specified	2
		C40-C41- Bone and joints	2
		C02- Tongue, other location not specified	1
		C03- Gum	1
		C06- Mouth, other location not specified	1
		C07- Parotid	1
		C09- Tonsil	1
		C15- Esophagus	1
		C16- Stomach	1
		C24- Extrahepatic bile duct	1
		C38- Pleura/mediastinum/hearth	1
		C50- Breast	1
		C60- Penis	1
		C64- Kidney without pelvis	1
		C66- Upper urinary tract	1
		C67- Urinary bladder	1
		C90- Myeloma and plasmacytoma	1
		C92- Myeloid leukemia	1
NHL(C83,C84, C85)**	88 (23.8)	C46- Kaposi sarcoma	23 (26.1)
		C83, C85- NHL***	15 (17.0)
		C81- Hodgkin lymphoma	9
		C34- Lung and bronchial	6
		C44- Skin carcinoma	6
		C21- Anus	4
		C22- Liver and intrahepatic bile duct	4
		C64- Kidney without pelvis	3
		C92- Myeloid leukemia	3
		C02- Tongue, other location not specified	2
		C61- Prostate	2
		C09- Tonsil	1
		C10- Oropharynx	1
		C15- Esophagus	1
		C16- Stomach	1
		C20- Rectum	1

		C26- Digestive organs, others locations poorly specified	1
		C62- Testis	1
		C66- Upper urinary tract	1
		C67- Urinary bladder	1
		C72- Spinal cord/cranial nerves	1
		C91- Lymphoid leukemia	1
Hodgkin lymphoma	14 (3.8)	C83-C85 NHL****	7
		C19- Rectosigmoid junction	1
		C21- Anus	1
		C34- Lung and bronchial	1
		C44- Skin carcinoma	1
		C46- Kaposi sarcoma	1
		C73- Thyroid	1
		C92- Myeloid leukemia	1
Anal cancer	12 (3.2)	Cancers of oral cavity	3
		-C02- Tongue, other location not specified	1
		-C03- Gum	1
		-C09- Tonsil	1
		C18- C20 Colon/rectum	2
		C43- Melanoma	1
		C44- Skin carcinoma	1
		C46- Kaposi sarcoma	1
		C50- Breast	1
		C61- Prostate	1
		C81- Hodgkin lymphoma	1
		C84- NK/T cell Lymphoma	1
Lung cancer	11 (3.0)	C18- Colon	2
		C34- Lung and bronchial	2
		C83-C84 NHL[†]	2
		C21- Anus	1
		C22- Liver and intrahepatic bile duct	1
		C30- Nasal cavity and middle ear	1
		C32- Larynx	1
		C43- Melanoma	1
Prostate cancer	10 (2.7)	C16- Stomach	2
		C67- Urinary bladder	2
		C20- Rectum	1
		C21- Anus	1
		C34- Lung and bronchial	1
		C43- Melanoma	1
		C44- Skin carcinoma	1
		C46- Kaposi sarcoma	1
Kidney cancer	10 (2.7)	C44- Skin carcinoma	2
		C83-C85 NHL[‡]	2
		C21- Anus	1
		C33- Trachea	1
		C34- Lung and bronchial	1
		C61- Prostate	1
		C67- Urinary bladder	1
		C74- Adrenal	1
Liver cancer	9 (2.4)	C83- Diffuse large B- cell lymphoma	2
		C06- Mouth, other location not specified	1
		C16- Stomach	1

		C22- Liver and intrahepatic bile duct	1
		C25- Pancreas	1
		C32- Larynx	1
		C34- Lung and bronchial	1
		C92- Myeloid leukemia	1
Upper aerodigestive cancers	19 (5.1)		
C02- Tongue, other location not specified	4	C01- Base of the tongue	3
		C34- Lung and bronchial	1
C03- Gum	2	C34- Lung and bronchial	1
		C41- Bone/joint, others location not specified	1
C04- Floor of the mouth	1	C46- Kaposi sarcoma	1
C05- Palate	2	C00- Lip	1
		C44- Skin carcinoma	1
C07- Parotid	1	C85- Others lymphoma not specified	1
C09- Tonsil	4	C02- Tongue, other location not specified	1
		C22- Liver and intrahepatic bile duct	1
		C34- Lung and bronchial	1
		C67- Urinary bladder	1
C12- Pyriform sinus	1	C10- Oropharynx	1
C14- Lip, oral cavity and pharynx locations poor specified	1	C85- Others lymphoma not specified	1
C32- Larynx	3	C34- Lung and bronchial	1
		C44- Skin carcinoma	1
		C61- Prostate	1

* Follicular lymphoma (C82):n= 1 ; Diffuse large B-cell lymphoma (C83): n= 13 ;NK/T cell lymphoma (C84): n= 3 ; Others lymphoma not specified (C85): n= 30; ** Diffuse large B-cell lymphoma (C83): n= 27; NK/T cell Lymphoma (C84): n= 1; Others lymphoma not specified (C85): n= 60; *** Others lymphoma not specified (C85):n= 5; Diffuse large B- cell lymphoma (C83): n= 10;**** Diffuse large B- cell lymphoma (C83): n= 5; Others lymphoma not specified (C85):n= 2. † Diffuse large B- cell lymphoma (C83) :n= 1 ; NK/T cell lymphoma (C84):n=1; ‡ Diffuse large B- cell lymphoma (C83) :n= 1; Others lymphoma not specified (C85):n=1; ¥SPCs :Second primary cancers, βMLWH: Men living with HIV.
